# Assessment of historical and future changes in temperature indices for winegrape suitability in Hungarian wine regions (1971–2100)

**DOI:** 10.3389/fpls.2025.1481431

**Published:** 2025-02-07

**Authors:** László Lakatos, Richárd Nagy

**Affiliations:** ^1^ Department of Environmental Science and Landscape Ecology, Eszterházy Károly Catholic University, Eger, Hungary; ^2^ Innoregion Knowledge Centre, Eszterházy Károly Catholic University, Eger, Hungary

**Keywords:** climate change, wine grape suitability, Average Growing Season Temperature (AGST), Growing Degree Days (GDD-Winkler index), Huglin index (HI), Biologically Effective Degree Days (BEDD)

## Abstract

Climate change is significantly impacting our agricultural crops and their cultivation areas, which are expected to change considerably by the end of the century. Temperature conditions decisively influence the safe suitability of grapes in a given location. To address these changes, we analysed the temporal changes of four temperature indicators: Average Growing Season Temperature (AGST), Growing Degree Days (GDD or Winkler index (GDD-WI), Huglin index (HI), and Biologically Effective Degree Days (BEDD) across 22 Hungarian wine regions from 1971 to 2100. The analysis was based on data from 14 climate models under RCP 4.5 and RCP 8.5 scenarios. To investigate the future suitability of wine grapes, we introduced the dynamic suitability function, which allowed us to analyse the suitability of the average temperature during the growing season for 21 wine grape varieties from 2031 to 2100 in decadal increments. Additionally, a temperature impact function was introduced to characterise the suitability of 21 wine grape varieties with values ranging from 0 to 1, based on the average temperature during the growing season. The results confirmed that the frequency of temperature indices used in grape cultivation will shift distinctly towards warmer climate classes in the future. The increasingly warmer climate presents certain advantages but also has growing cultivation risks. In the most optimistic scenario, the average temperature during the growing season may decrease by 0.8°C over the next seven decades. However, in the most pessimistic model, the change expected by the end of the century exceeds a 4.0°C increase. For wine grape varieties with lower heat requirements, suitability under the pessimistic RCP 8.5 emission scenario is projected to decrease by 29% by the end of the century. Conversely, under the optimistic scenarios, the decline in suitability values is only between 3-4%. For grape varieties with higher heat requirements, a 10% decrease in suitability is expected under the RCP 8.5 scenario. In contrast, the RCP 4.5 scenario suggests that suitability could improve by 1-2% by the end of the century. These findings contribute to a better understanding of the impacts and consequences of climate change and offer insights on how to prepare for these challenges in the viticulture sector.

## 1 Introduction

In light of accelerating global warming and its profound implications on agricultural practices, a critical evaluation of the temperature parameters that influence the suitability of grape cultivation has become imperative (Masson-Delmotte et al., 2021). Grapevines, being highly sensitive to climatic variables, especially temperature, are indicators of broader environmental changes (Jones et al., 2005). As such, understanding the thermal requirements and thresholds for viticulture is not only important for sustaining traditional wine regions but also for identifying new cultivation opportunities in a warming climate (Van Leeuwen and Darriet, 2016). In the past, the wine regions in the northern areas of Hungary represented the northern limit of viticulture due to climatic conditions. However, as a result of global warming, viticulture has expanded to regions significantly further north, such as Poland (Ziernicka-Wojtaszek and Zawora, 2007), the coastal areas of Germany (Schernewski, 2011), and Southern Scandinavia (Gustafsson and Mårtensson, 2005). To address these challenges, we examined the historical and projected future distributions of four key temperature indices across 22 Hungarian wine regions: Average Growing Season Temperature (AGST) (Jones, 2006a); the GDD-Winkler Index (GDD) (Winkler et al., 1974); the Huglin Index (HI) (Huglin, 1978); and Biologically Effective Degree Days (BEDD) (Gladstones, 1992).

### 1.1 Overview of Hungarian wine regions

In the past, the wine regions in the northern areas of Hungary represented the northern limit of viticulture due to climatic conditions. However, as a result of global warming, viticulture has expanded to regions significantly further north, such as Poland (Ziernicka-Wojtaszek and Zawora, 2007), the coastal areas of Germany (Schernewski, 2011), and Southern Scandinavia (Gustafsson and Mårtensson, 2005). Hungary is renowned for its diverse and historically significant wine regions, each offering unique terroirs influenced by varied climatic, geological, and topographical conditions. The country is home to 22 official wine regions, including internationally acclaimed areas such as Tokaj, Villány, and Eger.

- **Tokaj**: Situated in northeastern Hungary, Tokaj is a UNESCO World Heritage Site known for its Aszú wines, produced from noble rot-affected grapes. The region experiences a mix of continental and mild climate conditions (Szivas, 1999), with sufficient warmth during the growing season complemented by cool autumn nights (Szepesi et al., 2017).
- **Villány**: Located in the southern part of Hungary, Villány is the country’s warmest wine region, specializing in red wines, particularly Cabernet Franc. The Mediterranean influence ensures long, hot summers and mild winters, ideal for ripening full-bodied red grape varieties (Czigány et al., 2020).
- **Eger**: Positioned in northern Hungary, Eger is celebrated for its Egri Bikavér (Bull’s Blood) red blends and increasingly for its white varietals such as Leányka and Hárslevelű. Its temperate continental climate and diverse soil composition contribute to a broad range of grape suitability (Novák et al., 2023).

Other notable wine regions include Sopron, with its proximity to Lake Neusiedl providing a unique microclimate, and Balatonfüred-Csopak, located near Lake Balaton, where the moderating effects of the lake support the production of high-quality whites such as Olaszrizling (Németh et al., 2014).

These regions differ significantly in their microclimates, soil types, and historical viticultural practices, making Hungary an exceptional location for examining the impacts of climate change on grape suitability. Each region’s unique climatic conditions are essential for understanding how temperature shifts may influence grape phenology, yield, and quality (Szenteleki et al., 2012).

### 1.2 Global perspective on grapevine suitability

The safe cultivation of grapes is typically associated with Mediterranean climate zones, where favorable thermal conditions provide a stable environment for grape growth. Jackson (2008) emphasized that these regions are delimited by 10°C and 20°C isotherms, reflecting the lower and upper bounds of grapevine physiological tolerances. Schultz and Jones (2010) further refined this definition, suggesting that the average temperature during the growing season should range between 12°C and 22°C for optimal viticultural conditions. Similarly, Van Leeuwen and Seguin (2006) highlighted the importance of cumulative heat units above a base temperature of 10°C, proposing a threshold of 1000°C during the growing season for successful grape cultivation.

Temperature is a primary driver of grapevine phenological development, with annual temperature variations exerting a significant influence on the timing of key growth phases (Jones and Davis, 2000; Jones et al., 2005; Cook and Wolkovich, 2016; García de Cortázar-Atauri et al., 2017). Research has consistently demonstrated that temperature not only governs phenological events but also directly affects berry composition and quality. These findings underscore the preeminence of temperature over soil characteristics or varietal factors in determining grape development (Van Leeuwen et al., 2004; Jones, 2006b).

Climatic requirements are critical determinants of both the quantity and quality of grape yields (Santos et al., 2010). Grapes, as heat- and sunlight-demanding crops, require high solar radiation and elevated temperatures during the vegetative period and berry ripening stages (Malheiro et al., 2010). As global temperatures rise, wine regions face increasing challenges, including potential shifts in traditional cultivation zones. Dunn et al. (2019) and Tóth and Végvári (2016) highlighted the prospect of viticultural boundaries extending poleward in both hemispheres, creating new opportunities in countries like Poland (Kunicka-Styczyńska et al., 2016), Sweden (Rauhut Kompaniets, 2022), Canada (Jobin Poirier et al., 2021), and the United Kingdom (Gannon et al., 2023). At the same time, traditional wine-producing regions may confront heightened risks of unsuitability due to excessive heat or reduced cold tolerance (Duchêne et al., 2010).

### 1.3 Objectives of the study

Temperature is a key factor influencing grapevine development, phenology, and suitability for wine production. Understanding how temperature variables such as the GDD-Winkler Index, Growing Season Temperature, Biologically Effective Degree Days, and Huglin Index change over time is crucial for assessing the future adaptability of grapevine varieties to specific regions. This study examines the past and future distributions of these temperature variables across 22 Hungarian wine regions, following international best practices.

Our study aims to analyse how the distribution of the four essential temperature variables used to assess grape suitability (AGST, GDD, HI, BEDD) will change in the near and distant future. Furthermore, we sought to determine how the suitability of wine grapes can be quantitatively defined if the average temperature during the growing season is known. Based on the studies by Jones et al. (2005) and Hannah et al. (2013), we know the optimal temperature range for the suitability of many wine grape varieties (21 varieties). The cultivation risk increases whenever the average temperature during the growing season deviates from the specified values, either higher or lower. In our investigations, we determined the probability that the average temperature during the growing seasons from 2031 to 2100 would be optimal for the given grape variety, providing a comprehensive assessment of future cultivation conditions. Additionally, we sought to determine how much the cultivation risk for wine grape varieties changes if the average temperatures of the growing seasons increase, i.e., if the temperatures are lower or higher than the optimal temperature requirements of the given wine grape variety. The physiological processes of grapevines occur within the temperature range of 5–35°C (Gladstones, 1992; Mullins et al., 1992). This range provides the theoretical basis for assessing grapevine suitability under varying climatic conditions. Building on the research findings of Jones (2006a); Jones et al. (2012); Jones (2015), and Van Leeuwen and Darriet (2016), we developed an impact function to evaluate the adaptability of wine grape varieties to different growing conditions.

## 2 Materials and methods

The FORESEE 3.2 is a freely accessible database that contains daily data for seven meteorological variables (Dobor et al., 2014). It was developed based on the advanced error correction of daily maximum/minimum temperature and precipitation datasets from 10 regional climate models (RCMs) run under the ENSEMBLES European Union project (FP6). For precipitation, both its temporal distribution and amount were corrected. The corrected future climate projections were prepared using historical data from the E-OBS database. The impact of future human activities was considered using a medium scenario, the A1B SRES (Special Report on Emissions Scenarios; Nakicenovic et al., 2000) emission scenario.

### 2.1 Climate models used

The FORESEE 4.2 database incorporates data from 14 regional climate models of the EURO-CORDEX initiative, which adhere to the RCP4.5 and RCP8.5 scenarios and utilize a uniform bias correction methodology. Despite sharing these characteristics, the models differ in their physical parameterizations and climate projections. For example, IPSL-RCA4 exhibited the smallest bias in temperature and precipitation, while HadGEM2 projected the most significant summer droughts, highlighting the importance of ensemble-based assessments to capture uncertainties.

**Figure 1:**
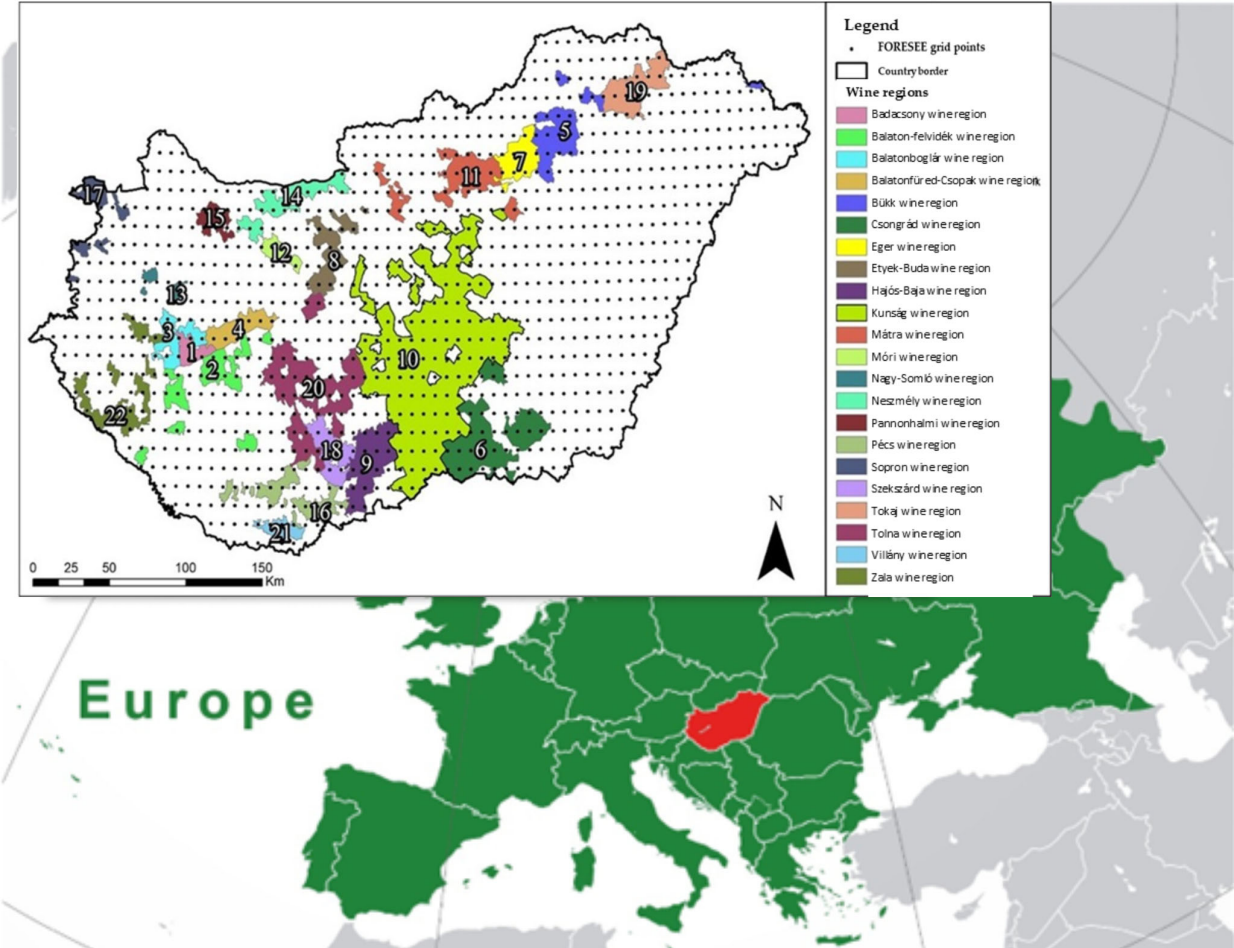
Geographical location of Hungary and its 22 wine regions. Reproduced from Lakatos & Mika (2022), published under the terms of the Creative Commons Attribution (CC BY) license (https://creativecommons.org/licenses/by/4.0/).

Additionally, to estimate the past and future climatic suitability of Hungarian wine regions, we utilized multiple datasets from the FORESEE system. The FORESEE-HUN v1.0 database (1971–2022) provided interpolated daily meteorological data at a 0.1° x 0.1° resolution, created using data from the Hungarian Meteorological Service (HUCLIM). Future projections were derived from FORESEE 4.0, which integrates 28 bias-corrected climate models, including the 14 models of the CORDEX experiment under the RCP4.5 and RCP8.5 scenarios (Kern et al., 2024). Using these datasets, we analyzed the distribution of four temperature indices (AGST, GDD-Winkler index, HI, BEDD index) across 22 Hungarian wine regions.The database covers the entire territory of Hungary and contains 2070 pixel data points per variable and model. The database specific to the 22 wine regions includes 300 pixels. The geographical location of Hungary and its 22 wine regions is illustrated in **Figure 1**, providing a visual representation of the study area and the spatial distribution of the wine regions analyzed in this study.

To evaluate the climatic suitability of grape cultivation in Hungarian wine regions, we focused on four temperature indices widely used in viticultural studies. These indices provide a quantitative framework for assessing the thermal conditions necessary for grapevine development, yield, and quality, and in this case, for quantitatively evaluating the suitability of viticulture. Below, we define these indices and explain their significance in the context of this study.

### 2.2 Definitions of key temperature indices

**AGST (Average Growing Season Temperature):**

- Represents the average temperature during the growing season, usually calculated from April to October (Jones, 2006a).

**GDD-WI (Growing Degree Days - Winkler Index):**

- Summarizes temperatures above a base temperature (10°C) during the growing season, typically between April and October (Winkler et al., 1974).

**HI (Huglin Index):**

- Summarizes the daily mean and maximum temperatures during the growing season, weighted by a latitude-dependent correction factor, calculated between April and September (Huglin, 1978).

**BEDD (Biologically Effective Degree Days):**

- Takes into account the effects of both daytime and nighttime temperatures by weighting and summing temperature variations in a biologically effective manner, calculated from April to October (Gladstones, 1992).

In this study, we examined the climatic suitability of grape cultivation across 22 official Hungarian wine regions, each characterized by distinct climatic, geological, and topographical conditions. Below, we provide an overview of the key grape varieties and their unique adaptations in the major wine regions.

To quantitatively evaluate the climatic suitability of Hungarian wine regions for different grape varieties, we developed a temperature-based suitability function. This approach allows us to assess how well the average growing season temperature (AGST) aligns with the optimal temperature requirements of each grape variety. Below, we present the methodology for calculating suitability values, which provides a numerical framework for comparing climatic conditions across regions and time periods.

### 2.3 Suitability calculation

The suitability values (S) were calculated using a temperature-based suitability function that evaluates the average growing season temperature (AGST) relative to the optimal temperature range of each grape variety. The suitability function S(T) is defined as follows:


$$S(T)=\{\begin{array}{cc} 0 & if\;T<T_{min}\;or\;T>T_{max}\\ \frac{T-T_{min}}{T_{opt,low}-T_{min}} & if\;T_{min}\leq T<T_{opt,low}\\ 1 & if\;T_{opt,low}\leq T\leq T_{opt,high}\\ 1-\frac{T-T_{opt,high}}{T_{max}-T_{opt,high}} & if\;T_{opt,high}<T\leq T_{max} \end{array}$$

Where:

- T_min_=5°C: The minimum threshold below which no suitability exists.
- T_max_=35°C: The maximum threshold above which no suitability exists.
- Topt,low: The lower bound of the optimal temperature range for a given grape variety.
- Topt,high: The upper bound of the optimal temperature range for a given grape variety.

This index provides a numerical value between 0 and 1, representing the degree of climatic suitability for a specific grape variety.

### 2.4 Hungarian wine regions and their grape varieties

This study examined the climatic suitability of 22 official Hungarian wine regions, each characterized by distinct climatic, geological, and topographical conditions. The regions represent a wide range of viticultural diversity, including unique grape varieties that have adapted to local conditions over centuries. Below is a detailed overview of the main grape varieties cultivated in the major wine regions included in the analysis:

**Tokaj Wine Region:**

o **Key Grape Varieties:** Furmint, Hárslevelű, Sárgamuskotály (Muscat Lunel)

o **Description:** Tokaj, located in northeastern Hungary, is world-renowned for its Aszú wines, primarily made from Furmint and Hárslevelű grapes affected by noble rot. These varieties thrive in volcanic soils and a microclimate characterized by warm summers and cool autumns, which are ideal for the slow ripening required for high-quality dessert wines (Fazekas et al., 2022).

**Villány Wine Region:**

o **Key Grape Varieties:** Kékfrankos, Cabernet Franc, Merlot, Cabernet Sauvignon

o **Description:** Villány, in southern Hungary, is the country’s warmest wine region. It specializes in red wines, with Cabernet Franc becoming the flagship variety. Long, hot summers and mild winters provide excellent conditions for full-bodied red grape varieties (Mód and Simon, 2012).

**Eger Wine Region:**

o **Key Grape Varieties:** Kékfrankos, Leányka, Hárslevelű, Olaszrizling

o **Description:** Situated in northern Hungary, Eger is celebrated for Egri Bikavér (Bull’s Blood), a red wine blend often based on Kékfrankos, and increasingly for its white varietals such as Leányka and Hárslevelű. The region’s diverse soils and temperate continental climate allow for a wide range of grape cultivation (Bálo et al., 2014).

**Sopron Wine Region:**

o **Key Grape Varieties:** Kékfrankos, Zweigelt, Pinot Noir

o **Description:** Located near Lake Neusiedl, Sopron benefits from a unique microclimate with relatively mild winters and cool summers. Kékfrankos dominates the region, producing vibrant, fruit-forward red wines (Németh et al., 2014).

**Balatonfüred-Csopak Wine Region:**

o **Key Grape Varieties:** Olaszrizling, Szürkebarát (Pinot Gris), Sauvignon Blanc

o **Description:** Positioned along the northern shore of Lake Balaton, this region is known for its high-quality white wines, particularly Olaszrizling. The lake’s moderating effects create a favorable climate for producing crisp, aromatic wines (Szabó and Závodi, 2018).

**Other Notable Regions:**

o **Mátra:** Known for its aromatic whites, such as Muscat Ottonel and Tramini.

o **Szekszárd:** Specializes in red blends similar to Eger, with Kadarka and Kékfrankos playing prominent roles.

o **Badacsony:** Famous for Olaszrizling and Kéknyelű, a unique variety found almost exclusively in this region (Hlédik and Harsányi, 2019).

**Grape Variety Adaptation and Climatic Considerations**

The grape varieties cultivated in these regions reflect the diverse climatic conditions of Hungary, ranging from the cool, continental climate of Tokaj to the Mediterranean-influenced climate of Villány (Hajdu, 2018; Czigány et al., 2020). This diversity makes Hungary an ideal study area for assessing how temperature shifts may impact grape phenology, yield, and quality ( (Fraga et al., 2012).Each grape variety has specific temperature thresholds for optimal ripening, which were factored into our analysis to evaluate future cultivation risks and opportunities (Van Leeuwen and Seguin, 2006).

We determined the values and distributions of the examined unique temperature indices, which are the Averege Growing Season Temperature (AGST), Growing Degree Days (GDD), Huglin Index (HI), Biologically Effective Degree Days (BEDD), and.

These indices were used to assess the suitability of wine grape varieties under different climatic scenarios.

The results are presented for three time periods used in the IPCC reports (IPCC, 2014). The reference values are characterized by the period from 1986 to 2005. Future changes relative to this period are presented for two time periods: the near future (2016-2035) and the distant future (2081-2100). In our analyses, we consistently follow these time scales. Additionally, we examine in detail how the heat requirements for 21 wine grape varieties will be met in the future. These are presented for 10-year periods, showing the probability that the average temperatures during the growing seasons fall within the specified optimum interval. Furthermore, we determined the probabilities of average temperatures falling below or above the optimum interval. Finally, based on previous research findings, we developed an impact function that allows us to estimate the cultivation suitability of wine grapes within the temperature range of 5-35°C.

The equations, class intervals, and names of the temperature indices used in the study are presented in **Table 1**.

A problem with using the GDD-Winkler index is that the Region I-V classification is not used in every wine-producing country. Therefore, it may be more appropriate to classify different growing regions into the following climate classes, as shown in **Table 2**.

**Table 1:** Names, equations, and class intervals of the temperature indices used in the study, including specific names of class intervals.

Variable	Equation	Class limits	
Growing Season Average Temperature AGST [°C]	$\sum_{Apr1}^{Oct\;31}\frac{[T_{max}-T_{min}]/2}{n}$	Too cool Cool Intermediate Warm Hot Very hot Too hot	<13°C 13–15°C 15–17°C 17–19°C 19–21°C 21–24°C >24°C
Growing Degree-Days GDD [°C]	$\sum_{Apr1}^{Oct\;31}max\{[\frac{\left(T_{max}-T_{min}\right)}{2}]-10\}$	Too cool (Region I) (Region II) (Region III) (Region IV) (Region V) Too hot	<850 850–1389 1389–1667 1667–1944 1944–2222 2222–2700 >2700
Huglin index (HI, C°)	$\sum_{Apr1}^{Sept\;30}max(\{[(T_{mean}-10)+(T_{max}-10)]/2\},0)*K$ where *K* is an adjustment for latitude/day length	Too cool Very cool Cool Temperate Warm temperate Warm Very warm Too hot	<1200 1200–1500 1500–1800 1800–2100 2100–2400 2400–2700 2700–3000 >3000
Biologically effective degree-days (BEDD, C°)	$\sum_{Apr1}^{Oct\;31}min[(max\{[\frac{\left(T_{max}-T_{min}\right)}{2}]-10,0\}*K+DTR_{adj}),9]$ where $DTR_{adj}=\{\begin{array}{c} 0.25[DTR-13]\;,[DTR]\;>13\\ 0,\;10<[DTR]<13\\ 0.25\;[DTR-10],\;[DTR]<10 \end{array}\}$ Where *K* is an adjustment for latitude/day length	Too cool Too hot	<1000 1000–1200 1200–1400 1400–1600 1600–1800 1800–2000 2000–2200 >2200

Average Growing Season Temperature (AGST) classification is based on Jones (2006a), Growing Degree Days (GDD) classification follows Winkler et al. (1974), Huglin Index (HI) is adapted from Huglin (1978), and Biologically Effective Degree Days (BEDD) follows Gladstones (1992).

**Table 2:** Classification of traditional viticultural regions into different climatic classes based on GDD-Winkler index values.

GDD-Winkler (values)	Types of Climate
Too cool (GDD<850°C)	Too cool
Region I (850°C<GDD<1389°C)	Cool
Region II (1389°C<GDD<1667°C)	Intermediate
Region III (1667°C<GDD<1944°C)	Warm
Region IV (1944°C<GDD<2222°C)	Hot
Region V (2222°C<GDD<2700°C)	Very hot
Too hot (GDD>2700°C)	Too hot

The classification presented in this table was developed by the authors. It is analogous to the climate categories proposed by Jones (2006a) for average growing season temperatures, adapted here to the GDD-Winkler index values to enhance interpretability.

### 2.5 Dynamic AGST function for temporal analysis

To illustrate temporal changes in AGST, we introduced the Dynamic AGST function or DAGST function. This method allows us to track decade-by-decade how the average temperature of the growing season will change in the future and how this relates to the optimal temperature requirements of the 21 examined grape varieties. By considering the most pessimistic case for the 22 Hungarian wine regions and the 14 examined models, using data from the two climate scenarios (RCP4.5 and RCP8.5), we can assess whether all Hungarian growing sites will remain suitable for cultivating the main grape varieties in the future.

### 2.6 Optimal heat requirements of wine grapes

For 21 wine grape varieties, optimal temperature ranges were determined using the functional approach of Jones et al. (2005) and the numerical values provided by Hannah et al. (2013). These ranges were used to create class intervals for each variety, allowing for the analysis of the likelihood that the average temperatures during the growing seasons fall within the specified optimum intervals.

## 3 Result

### 3.1 Overview of bioclimatic indicators and general trends

We analysed the frequencies of the four climatic variables studied over three periods. The class intervals with the highest frequencies show a clear shift towards warmer intervals in the future (**Table 3**). Based on AGST, Hungarian wine regions have predominantly belonged to the “warm” range in the past; however, in the future, we can expect the most frequent occurrences to be classified as “hot” vintages. This indicates a significant impact on the thermal suitability of grape varieties across Hungarian wine regions, emphasizing the necessity for adaptive strategies. The GDD-Winkler index shows a similar warming trend, with historical “intermediate” conditions (Region II) transitioning to “warm” vintages (Region III) in the near future and predominantly “hot” vintages (Region IV) by the end of the century.

**Table 3:** Distribution of frequency of occurrence of different climatic class intervals for the four temperature indices.

AGST	1986-2005	2016-2035	2081-2100	GDD-Winkler	1986-2005	2016-2035	2081-2100
Too cool(AGST<13°C)	0.1	0.1	0.0	Too cool (GDD<850°C)	0.1	0.1	0.0
Cool (13°C<AGST<15°C)	2.0	1.6	0.1	Cool (850°C<GDD<1389°C)	10.3	7.9	0.5
Intermediate(15°C<AGST<17°C)	35.5	20.0	1.8	termediate(1389°C<GDD<1667°C)	56.7	31.3	3.9
Warm (17°C<AGST<19°C)	62.3	60.1	23.1	Warm (1667°C<GDD<1944°C)	32.6	40.6	19.6
Hot (19°C<AGST<21°C)	0.1	17.5	51.0	Hot(1944°C<GDD<2222°C)	0.4	18.2	37.9
Very hot (21°C<AGST<24°C)	0.0	0.8	23.3	Very hot (2222°C<GDD<2700°C)	0.0	1.9	33.8
Too hot (AGST>24°C)	0.0	0.0	0.7	Too hot (GDD>2700°C)	0.0	0.0	4.2
**a**				**b**			
Huglin-Index (HI)	1986-2005	2016-2035	2081-2100	BEDD	1986-2005	2016-2035	2081-2100
Too cool (HI<1200)	0.0	0.0	0.0	Too cool (BEDD<1000)	1.2	1.0	0.0
Very cool (1200<HI<1500)	0.7	0.4	0.0	1000<BEDD<1200	8.2	8.3	0.7
Cool (1500<HI<1800)	3.8	3.8	0.2	1200<BEDD<1400	73.9	46.8	10.1
Temperate (1800<HI<2100)	46.0	23.5	2.5	1400<BEDD<1600	16.7	42.9	61.4
Warm temperate (2100<HI<2400)	40.9	43.8	17.6	1600<BEDD<1800	0.0	1.0	27.1
Warm (2400<HI<2700)	8.7	24.6	39.4	1800<BEDD<2000	0.0	0.0	0.7
Very warm (2700<HI<3000)	0.0	3.4	27.9	2000<BEDD<2200	0.0	0.0	0.0
Too hot (HI>3000)	0.0	0.4	12.4	Too hot (BEDD>2200)	0.0	0.0	0.0
**c**				**d**			

a) The table shows the distribution function of AGST class intervals across the three examined periods..

b) The table shows the distribution function of GDD-Winkler index class intervals across the three examined periods.

c) The table shows the distribution function of Huglin index class intervals across the three examined periods.

d) The table shows the distribution function of BEDD index class intervals across the three examined periods.

The yellow highlights in **Table 3** indicate the class intervals with the highest frequencies across the three examined periods. These highlights are used to emphasize the most significant ranges within the dataset.

The Huglin index supports these findings, showing that historically “temperate” regions will shift to “warm temperate” or even “warm” conditions in the future, reflecting global warming’s impact on viticulture. Similarly, BEDD values are projected to increase, with the most frequent range shifting from 1200–1400°C in the past to 1400–1600°C in both the near future and the distant future. In the near future, and even more so in the distant future, we can expect the BEDD sums during the growing season to range between 1400–1600°C.

### 3.2 Spatial patterns and regional differences

**Figure 2:**
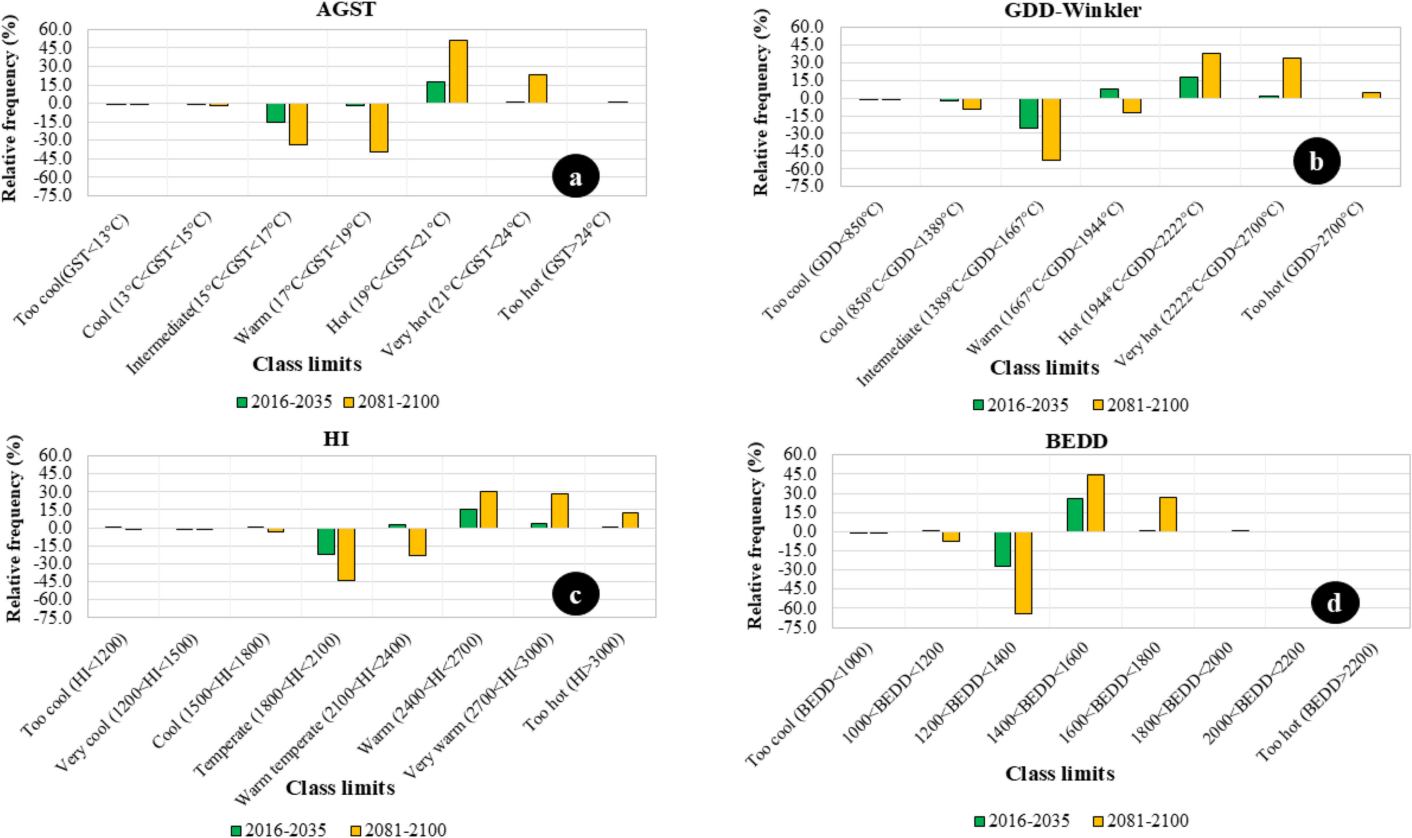
Changes in the Four Examined Temperature Variables Distribution Compared to the Historical Reference Period of 1986-2005. The following figures show the changes in the distribution of the four examined temperature parameters compared to the historical reference period of 1986-2005. **(A)** shows the change in the distribution of AGST class intervals compared to the historical reference period of 1986-2005. **(B)** shows the change in the GDD-Winkler index compared to the historical reference period of 1986-2005. **(C)** shows the change in the distribution of Huglin index class intervals compared to the historical reference period 1986-2005. **(D)** shows the change in the distribution of BEDD index class intervals compared to the historical reference period of 1986-2005.

We analysed the distribution of the studied temperature variables that will change in the near and distant future compared to the recent past (1986-2005) as a reference period. The following observations can be made:

For AGST, the occurrence frequencies of the “intermediate” and “warm” climatic class intervals will decrease in the near future. However, the decrease will not exceed 15%. However, in the distant future, the decrease will exceed 30%. The frequency of “hot” climatic vintages will increase by more than 15% in the near future. In contrast, in the distant future, the “hot” climatic class interval will occur 50% more frequently than in the past. Additionally, in the distant future, the frequency of “very hot” vintages will increase by more than 20% compared to historical data (**Figure 2A**).

The GDD-Winkler index also supports the prediction of definite future warming. In Hungarian wine regions, the occurrence frequency of “intermediate” climatic conditions, characteristic of Region II, will decrease by more than 25% in the near future. In contrast, the frequency of “hot” climatic vintages, characteristic of Region IV, will increase by more than 15%. These changes will significantly intensify in the distant future compared to historical data. The probability of “intermediate” climatic vintages, characteristic of Region II, will decrease by more than 50%.

In contrast, the occurrence of “warm” vintages, characteristic of Region III, will decrease by 15%. Conversely, the occurrence of “hot” climatic vintages, characteristic of Region IV, will increase by more than 35%. The occurrence of “very hot” climatic vintages, characteristic of Region V, will increase by 30% in the distant future (**Figure 2B**).

In the near future, the frequency of HI values characteristic of “temperate” climatic areas will decrease by more than 20%. Compared to past values, “warm” climatic effects will increase by more than 15%. In the distant future, the frequency of “temperate” climatic vintages will decrease by more than 40%, and the probability of “warm temperate” vintages will decrease by more than 20%. Meanwhile, the likelihood of “warm” vintages will increase by more than 30%, “very warm” vintages by more than 25%, and the probability of “too hot” vintages will increase by more than 10% in the distant future (**Figure 2C**).

In the near future, the occurrence frequency of vintages with a BEDD between 1200 and 1400 will decrease by more than 25%, while the probability of vintages with a BEDD between 1400 and 1600 will increase by more than 25%. In the distant future, these changes will intensify: the occurrence frequency of vintages with a BEDD between 1200 and 1400 will decrease by more than 60%, while the probability of vintages with a BEDD between 1400 and 1600 will increase by more than 40%, and the likelihood of vintages with a BEDD between 1600 and 1800 will increase by more than 25% (**Figure 2D**).

**Figure 3:**
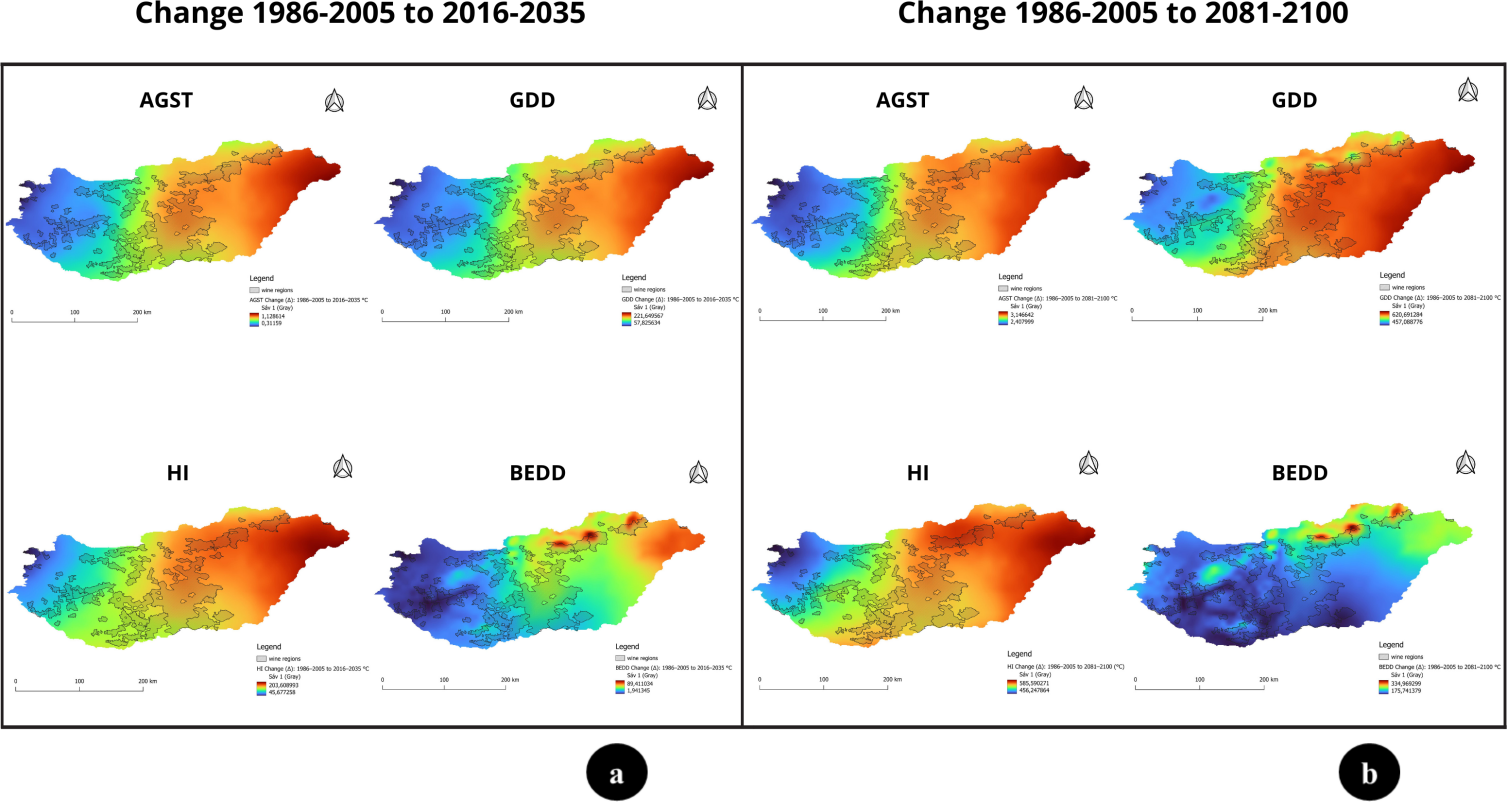
Changes in the distribution of temperature indices (AGST, GDD, HI, BEDD) in Hungary’s wine regions between 1986–2005 and 2016–2035 **(A)**, as well as between 1986–2005 and 2081–2100 **(B)**.

In the following, we will provide a detailed analysis of how the spatial patterns of the four examined temperature variables (AGST, GDD, HI, and BEDD) have changed in Hungary’s wine regions compared to the 1986–2005 baseline period. The changes are presented for a near future period (2016–2035) and a distant future period (2081–2100) (**Figure 3**).

The exploration of spatial differences can play a crucial role not only in shaping the future viticultural strategies of the wine regions but also in influencing the economic and cultural development trajectories of these areas. These data help us better understand how individual wine regions will be able to adapt to the new conditions brought about by climate change and to what extent they will be able to do so.”

**Figure 3A** illustrates the regional distribution of changes in temperature indices (AGST, GDD, HI, BEDD) across Hungary’s wine regions compared to the reference period 1986–2005.

- **AGST (Average Growing Season Temperature):** Changes in AGST range from 0.36°C (Sopron wine region) to 0.91°C (Tokaj wine region), with a national average of 0.64°C across the 22 wine regions.
- **GDD (Growing Degree Days):** GDD changes range from 66.16°C (Sopron wine region) to 173.96°C (Tokaj wine region), with a national average of 122.48°C.
- **HI (Huglin Index):** Changes in the Huglin Index range from 56.87°C (Sopron wine region) to 170.62°C (Eger wine region), with an average increase of 124.56°C.
- **BEDD (Biologically Effective Degree Days):** Changes in BEDD range from 4.79°C (Sopron wine region) to 60.02°C (Tokaj wine region), with a national average of 24.87°C.

Overall, the data suggest that changes in temperature indices tend to increase more significantly in eastern wine regions compared to western regions.

**Figure 3B** illustrates the regional distribution of changes in temperature indices (AGST, GDD, HI, BEDD) across Hungary’s wine regions compared to the reference period 1986–2005 for the distant future.

- **AGST (Average Growing Season Temperature):** AGST changes range from 2.44°C (Sopron wine region) to 2.96°C (Tokaj wine region), with a national average of 2.71°C across the 22 wine regions.
- **GDD (Growing Degree Days):** GDD changes range from 475.98°C (Sopron wine region) to 581.65°C (Kunság wine region), with a national average of 537.23°C.
- **HI (Huglin Index):** Huglin Index changes range from 461.58°C (Sopron wine region) to 566.15°C (Eger wine region), with an average increase of 523.8°C.
- **BEDD (Biologically Effective Degree Days):** Changes in BEDD range from 182.71°C (Szekszárd wine region) to 258.49°C (Bükk wine region), with a national average of 204.26°C.

Unlike the near future (2016–2035), where regional differences were more pronounced, the distant future (2081–2100) shows a tendency for decreased regional disparities in AGST, GDD, and HI. However, BEDD exhibits a contrasting trend, with its differences increasing between regions. Northern wine regions also tend to experience stronger warming compared to southern ones.

### 3.3 Temporal changes in AGST based on RCP4.5 and RCP8.5 scenarios

For the 22 Hungarian wine regions, we produced projections for seven decades, covering the period from 1931 to 2100, showing how the AGST values will change in the future based on the most pessimistic, optimal, and average of the 14 models. The most pessimistic change refers to the model result that shows the highest temperature increase over the seven decades across the 22 wine regions. The most optimal change refers to the model result that shows the smallest temperature increase from 1931 to 2100 across the 22 wine regions. Under the RCP4.5 scenario, this could even mean a decrease.

For the RCP4.5 scenario, the HadGEM2-CCLM model was the most pessimistic for the Hajós-Baja wine region. At the same time, the most optimistic change was seen with the NCC-HIRHAM5 model in the Kunság wine region. In this case, the most optimal change represents a decrease, with the AAGST value during the growing season decreasing by 0.8°C over seven decades. The observed differences in AGST projections between the most optimistic, the most pessimistic, and the ensemble average models during the 2031–2040 period can be attributed to several factors. Firstly, the early projection period is particularly sensitive to systematic biases caused by the transition from observation-based historical data to modeled projections. Secondly, internal climate variability has a stronger influence on shorter periods, such as a single decade, amplifying model differences. Additionally, variations in regional parameterization and model sensitivity to greenhouse gas emissions contribute to these deviations. These factors emphasize the importance of ensemble averages for providing balanced and robust projections.”

**Figure 4:**
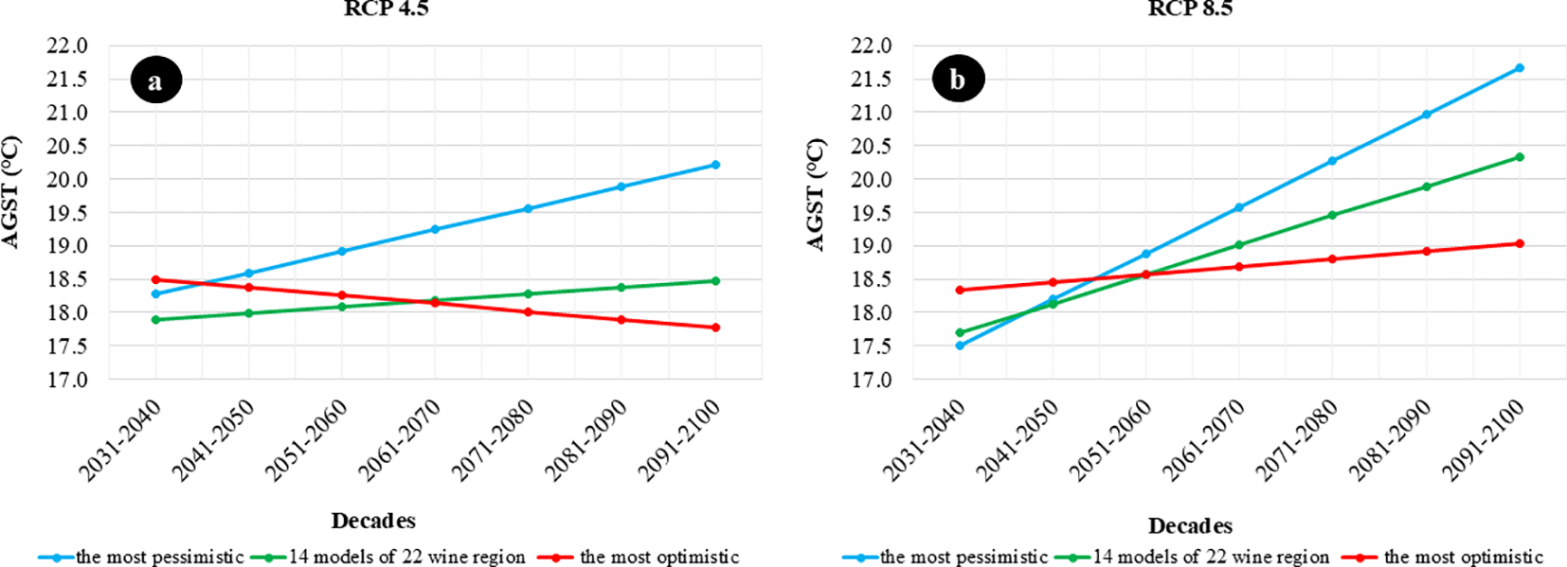
Decadal Trends of the Most Pessimistic, Average, and Most Optimistic AGST Change from 1931 to 2100. **(A)** shows the decadal trend of the most pessimistic, average, and most optimistic AGST changes from 1931 to 2100 based on the RCP4.5 scenario. **(B)** shows the decadal trend of the most pessimistic, average, and most optimistic AGST changes from 1931 to 2100 based on the RCP8.5 scenario.

However, even under the RCP4.5 scenario, the most pessimistic model shows an increase of nearly 2.0°C (**Figure 4A**).

Under the RCP8.5 scenario, the HadGEM2-RACMO22E model was the most pessimistic for the Sopron wine region. In contrast, the MPI-REMO2009 r1 model showed the most optimistic AGST change in the Balatonfüred-Csopak wine region. For the most optimistic change, the average AGST value during the growing season increases by 1.0°C over seven decades. However, the most pessimistic model indicates an increase of more than 4.0°C (**Figure 4B**).

### 3.4 Changes in AGST and the relationship between the dynamic AGST function and the suitability of grape varieties

The studies by Jones (2006a), Van Leeuwen et al. (2013), and Hannah et al. (2013) predominantly describe a static correlation between the Averege Growing Season Temperature (AGST) and the suitability of grapevine varieties. These studies highlight the AGST values for specific locations over a defined period, juxtaposed with the average heat requirement ranges of key grapevine varieties. Each grapevine variety is optimally cultivated within a defined temperature range, beyond which its viability is compromised. Specifically, if the mean AGST at a given location falls below or exceeds the temperature range required by a particular grape variety, the cultivation of that variety is deemed unsuitable for the site.”

**Figure 5:**
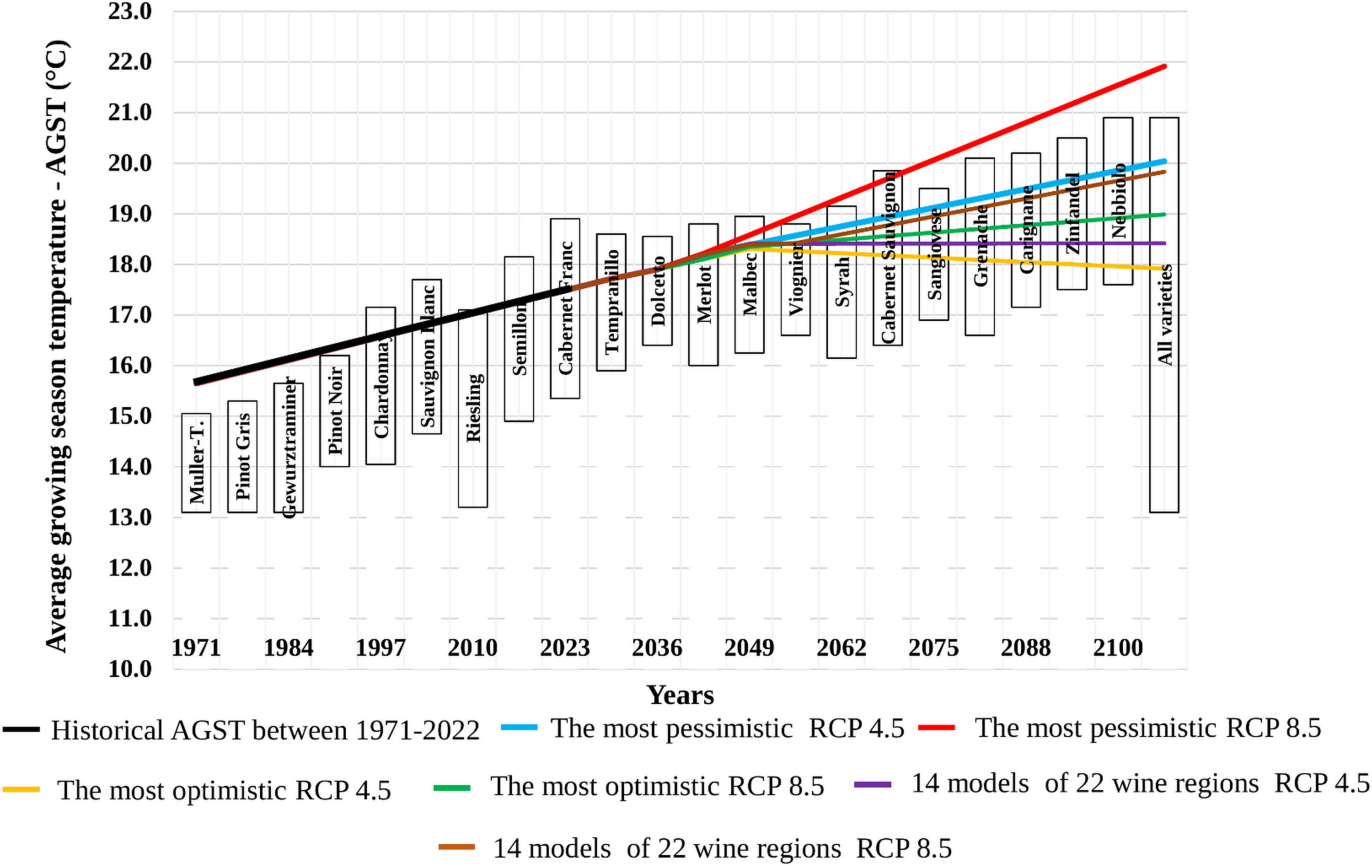
The figure presents the relationship between grapevine suitability and the average growing season temperature (AGST), illustrating the temporal changes in DAGST based on three scenarios: the most pessimistic (PDAGST), the average (ADAGST), and the most optimistic (ODAGST). The temporal change consists of two phases: the first phase includes historical measured temperature data (1971–2020), while the second phase presents modeled data for the 22 Hungarian wine regions under the most pessimistic, average, and most optimistic RCP4.5 and RCP8.5 scenarios.

The Dynamic Average Growing Season Temperature (DAGST) calculation method allows us to track decade-by-decade changes in the average temperature of the growing season in the future and assess how these relate to the optimal temperature requirements of the 21 examined grapevine varieties. Using data from 22 Hungarian wine regions and 14 climate models, along with two radiative forcing scenarios (RCP4.5 and RCP8.5), we can analyze how the average temperature of the growing season evolves under the most optimistic and most pessimistic scenarios. Furthermore, we can evaluate whether, according to the 14 model results, all Hungarian growing sites will remain suitable for cultivating the main grapevine varieties in the future. Based on the three scenario types (most optimistic, most pessimistic, and the 14 climate model results) and the two radiative forcing scenarios (RCP4.5 and RCP8.5), we can illustrate the progression of six DAGST functions for the period 2031–2100, alongside the optimal temperature ranges of 21 grapevine varieties (**Figure 5**).

The results highlight the following:

1. Pessimistic Scenario (RCP8.5): Under this scenario, the average temperature during the growing season exceeds 21°C in all Hungarian wine regions by the end of the century, surpassing the maximum temperature requirement of the most heat-intensive grape variety, Nebbiolo. This indicates that only grape varieties with very high heat requirements will be safely cultivable in these regions.
2. Average Model Assessment: Based on the average of the 14 climate models, nearly all grapevine varieties remain safely cultivable under both RCP4.5 and RCP8.5 scenarios. From the growers’ perspective, the temperature conditions will become favourable for cultivating higher heat-demanding varieties that were not suitable previously.
3. Optimistic Scenario (RCP4.5): According to the most optimistic climate model, the AGST values increase until the mid-2030s and then slightly decline by the end of the century. The magnitude of the decline is almost equal to the initial increase, resulting in average growing season temperatures similar to current values. Under this scenario, even the most heat-demanding grape varieties will remain safely cultivable in the coldest Hungarian wine regions.

### 3.5 Fulfilment of heat requirements for wine grape varieties

**Figure 6:**
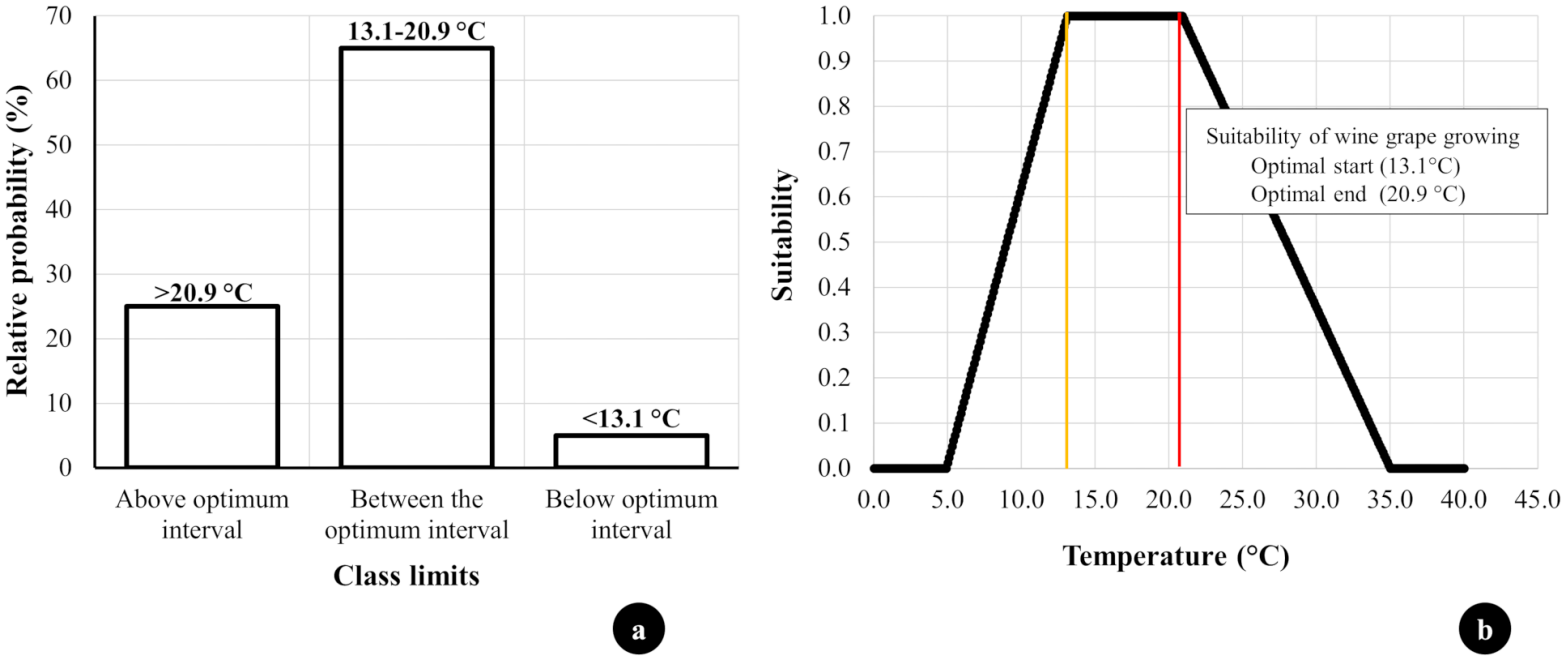
**(A)** The Probability Values of Heat Requirements for the 21 Examined Wine Grape Varieties in Hungarian Wine Regions. **(B)** Impact Function of Average Temperature During the Vegetation Period on the Suitability of 21 Grape Varieties The impact function of the average temperature during the vegetation period on the suitability of the 21 grape varieties studied. The range of interpretation of the impact function is between 5-35°C. The 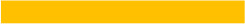 line shows the lower value of the optimum temperature range. In contrast, the 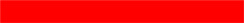 line shows the upper value of the optimum temperature range.

Knowing the optimal temperature requirements of the 21 examined wine grape varieties, we calculated the probability that the average temperatures during the growing seasons will fall within the optimal intervals specified for each variety over 10-year periods. Additionally, we determined the probabilities of average temperatures falling below or above the optimal intervals (**Figure 6A**). These probabilities were calculated for three different temperature class intervals from 2031 to 2100 under two emission scenarios (RCP 4.5 and RCP 8.5).

However, claiming that these grape varieties could not be cultivated in such years would be an exaggeration. In practice, grape varieties are often cultivated beyond their optimal temperature ranges, even when the long-term average temperature of the growing season exceeds their preferred range. We also cultivate grape varieties whose optimal temperature range is lower than the current long-term average temperature of the growing season.”

The probability of occurrence within the optimal temperature interval generally decreases for most of the 21 examined grape varieties from the near future (2031-2040) to the distant future (2091-2100) (**Table 4**). However, for the high heat-demanding varieties such as Carignane, Zinfandel, and Nebbiolo, there is an observed increase in the frequency of optimal temperatures towards the distant future under the optimistic RCP 4.5 scenario.

**Table 4:** Temporal changes in the probability of occurrence within the optimal temperature interval for 21 wine grape varieties over decades from 2021 to 2100, based on RCP 4.5 and RCP 8.5 scenarios.

Grape varieties	2031-2040		2041-2050		2051-2060		2061-2070		2071-2080		2081-2090		2091-2100	
	RCP4.5	RCP8.5	RCP4.5	RCP8.5	RCP4.5	RCP 8.5	RCP4.5	RCP 8.5	RCP4.5	RCP 8.5	RCP4.5	RCP 8.5	RCP4.5	RCP8.5
**Muller-Thurgau**	1,27	1,04	0,99	0,95	1,03	0,65	0,79	0,39	0,88	0,28	0,76	0,10	0,71	0,05
**Pinot Gris**	1,72	1,39	1,34	1,33	1,45	0,94	1,05	0,49	1,26	0,34	1,00	0,17	0,93	0,08
**Gewurztraminer**	2,72	2,26	2,05	2,16	2,25	1,35	1,59	0,73	2,05	0,54	1,44	0,27	1,32	0,15
**Pinot Noir**	6,15	4,79	4,47	4,73	5,08	2,70	3,15	1,26	4,49	0,90	2,83	0,47	2,45	0,32
**Chardonnay**	24,81	19,39	16,94	18,53	17,49	10,57	13,92	4,83	14,92	2,80	14,43	1,32	11,51	0,94
**Sauvignon Blanc**	43,05	35,58	32,33	32,56	32,22	19,74	26,99	11,29	26,84	6,43	28,26	2,67	24,64	1,76
**Riesling**	23,58	18,36	16,25	17,70	16,60	9,99	13,07	4,59	14,22	2,59	13,46	1,25	10,85	0,90
**Semillon**	58,44	50,50	48,34	47,19	46,28	31,11	40,98	19,57	40,04	13,24	41,21	5,34	38,78	3,22
**Cabernet Franc**	79,62	72,26	73,60	69,38	70,91	56,77	63,87	39,70	68,18	31,52	63,61	15,97	60,82	9,46
**Tempranillo**	70,43	62,72	62,10	59,05	59,36	44,81	53,88	30,24	55,01	22,94	54,27	10,39	51,61	5,89
**Dolcetto**	64,80	58,14	57,31	54,47	54,13	41,28	50,40	28,21	50,22	21,32	51,03	9,35	48,67	5,31
**Merlot**	75,01	67,89	68,65	64,53	65,70	52,14	59,76	36,03	62,62	28,36	59,83	13,76	57,18	7,96
**Malbec**	76,64	70,15	72,20	67,46	68,81	56,94	63,24	40,54	66,68	32,58	63,12	16,77	60,64	10,03
**Viognier**	69,23	63,23	64,12	59,82	61,14	49,54	56,67	35,05	58,45	27,79	56,78	13,44	54,89	7,73
**Syrah**	81,46	75,49	78,72	73,88	75,08	63,93	68,94	47,28	73,87	39,24	68,78	21,38	66,32	13,51
**Cabernet Sauvignon**	88,64	87,71	90,22	85,67	85,79	80,96	83,97	71,55	85,64	59,47	82,63	39,96	80,60	29,42
**Sangiovese**	76,53	75,75	79,19	74,33	75,28	69,36	71,77	58,09	75,05	49,43	71,34	29,54	70,44	20,40
**Grenache**	87,89	88,69	90,12	85,97	87,20	84,27	87,03	77,71	87,27	64,97	85,29	47,51	83,31	36,56
**Carignane**	76,23	80,19	82,69	77,77	80,37	80,77	81,51	77,59	82,03	65,82	79,42	50,14	78,85	39,14
**Zinfandel**	66,14	72,40	75,51	71,70	74,29	78,13	76,44	79,91	77,94	70,53	74,07	58,12	76,15	48,14
**Nebbiolo**	63,29	70,18	73,52	71,73	72,99	78,89	75,88	83,59	77,08	78,30	74,27	67,73	77,47	59,66
**All varieties**	99,74	99,56	99,76	99,04	99,71	95,26	97,99	92,09	99,32	83,01	97,82	69,77	96,83	61,06

This table shows the probability values for every ten years, indicating the likelihood that the average temperatures during the growing seasons will fall within the optimal intervals for each of the examined grape varieties. The analysis helps to understand the potential impacts of climate change on grape cultivation under varying climatic conditions.

### 3.6 Suitability of average temperature during the vegetation period for viticulture

The physiological processes of grapevines occur within the temperature range of 5–35°C (Gladstones, 1992; Mullins et al., 1992). Utilizing and further developing the research findings published by Jones (2006c); Keller (2010); Jones et al. (2012); Jones (2015), and Van Leeuwen and Darriet (2016), we created an impact function. This function calculates a value between 0 and 1 within the 5–35°C range, based on the average temperature during the growing season (AGST), to quantitatively assess the suitability of wine grape varieties (**Figure 6B**).

The calculated suitability values S(T) provide a quantitative measure of climatic suitability for grape varieties under varying temperature conditions. Historical data show that most varieties in Hungarian wine regions exhibit S-values within the optimal range, consistent with current viticultural practices. For future scenarios, high S-values (above 0.8) align with observations from other regions, where successful grape cultivation persists despite average growing season temperatures exceeding the optimal range (e.g., Burgundy and the Rhone Valley) (Van Leeuwen et al., 2013). It should be noted that the suitability index does not explicitly define what specific S-values (e.g., 0.95, 0.9, 0.85) correspond to in terms of cultivation security or yield quality. These interpretations require additional empirical data and validation, which were beyond the scope of the current study. Future research could focus on linking S-values to specific levels of production risk or quality assurance.The decadal changes in suitability values calculated by the introduced temperature impact function for grape varieties are shown in **Table 5**. The results indicate that for lower heat-requirement grape varieties, such as Muller-Thurgau, the suitability value does not drop below 0.71 from the current 0.83 by the end of the century, even under the pessimistic RCP 8.5 scenario. Under optimistic scenarios, the decrease in suitability values for lower heat-requirement grape varieties is a maximum of 3-4%, representing a relatively small change. Meanwhile, for higher heat-requirement varieties, such as Zinfandel or Nebbiolo, the temperature suitability for cultivation may improve by 1-2%.

**Table 5:** Decadal changes in suitability values calculated by the introduced temperature impact function for the period 2021-2100 based on RCP 4.5 and RCP 8.5 scenarios for 21 grape varieties.

Grape varieties	2031-2040		2041-2050		2051-2060		2061-2070		2071-2080		2081-2090		2091-2100	
	RCP4.5	RCP 8.5	RCP4.5	RCP 8.5	RCP4.5	RCP 8.5	RCP4.5	RCP 8.5	RCP4.5	RCP 8.5	RCP4.5	RCP 8.5	RCP4.5	RCP 8.5
**Muller-Thurgau**	0,84	0,83	0,83	0,83	0,83	0,80	0,81	0,78	0,82	0,76	0,81	0,72	0,81	0,71
**Pinot Gris**	0,87	0,86	0,86	0,85	0,85	0,83	0,84	0,80	0,85	0,78	0,84	0,75	0,83	0,73
**Gewurztraminer**	0,88	0,87	0,87	0,87	0,87	0,84	0,86	0,82	0,86	0,80	0,86	0,77	0,85	0,75
**Pinot Noir**	0,91	0,90	0,90	0,89	0,89	0,87	0,88	0,84	0,89	0,82	0,88	0,79	0,87	0,77
**Chardonnay**	0,95	0,94	0,94	0,93	0,93	0,91	0,92	0,88	0,93	0,86	0,92	0,83	0,91	0,81
**Sauvignon Blanc**	0,97	0,96	0,96	0,95	0,96	0,93	0,95	0,91	0,95	0,89	0,94	0,85	0,94	0,83
**Riesling**	0,93	0,92	0,92	0,92	0,92	0,89	0,91	0,87	0,91	0,84	0,90	0,81	0,90	0,79
**Semillon**	0,98	0,97	0,97	0,97	0,97	0,95	0,96	0,93	0,97	0,91	0,96	0,88	0,96	0,85
**Cabernet Franc**	0,99	0,99	0,99	0,99	0,99	0,98	0,98	0,96	0,99	0,94	0,98	0,91	0,98	0,89
**Tempranillo**	0,99	0,98	0,98	0,98	0,98	0,97	0,97	0,95	0,98	0,93	0,97	0,90	0,97	0,88
**Dolcetto**	0,98	0,98	0,98	0,98	0,98	0,96	0,97	0,95	0,97	0,93	0,97	0,89	0,97	0,87
**Merlot**	0,99	0,99	0,99	0,98	0,98	0,97	0,98	0,96	0,98	0,94	0,98	0,91	0,97	0,89
**Malbec**	0,99	0,99	0,99	0,98	0,98	0,97	0,98	0,96	0,98	0,94	0,98	0,91	0,98	0,89
**Viognier**	0,98	0,98	0,98	0,98	0,98	0,97	0,98	0,96	0,98	0,94	0,98	0,91	0,97	0,89
**Syrah**	0,99	0,99	0,99	0,99	0,99	0,98	0,99	0,97	0,99	0,95	0,98	0,92	0,98	0,91
**Cabernet Sauvignon**	0,99	0,99	0,99	0,99	0,99	0,99	0,99	0,98	0,99	0,97	0,99	0,95	0,99	0,93
**Sangiovese**	0,99	0,99	0,99	0,99	0,99	0,98	0,99	0,98	0,99	0,96	0,99	0,94	0,98	0,92
**Grenache**	0,99	0,99	0,99	0,99	0,99	0,99	0,99	0,99	0,99	0,98	0,99	0,96	0,99	0,95
**Carignane**	0,98	0,99	0,99	0,99	0,99	0,99	0,99	0,99	0,99	0,98	0,99	0,96	0,99	0,95
**Zinfandel**	0,98	0,98	0,98	0,98	0,98	0,99	0,99	0,99	0,98	0,98	0,99	0,97	0,99	0,96
**Nebbiolo**	0,97	0,98	0,98	0,98	0,98	0,99	0,98	0,99	0,98	0,99	0,98	0,98	0,99	0,97
**All varieties**	1,00	1,00	1,00	1,00	1,00	1,00	1,00	1,00	1,00	0,99	1,00	0,98	1,00	0,97

A value of 1 indicates that the given grape variety can be grown entirely safely. In contrast, a value of 0 indicates that the given grape variety cannot be grown based on the average temperature of the vegetation period.

### 3.7 Projected temperature changes in hungarian wine regions based on different emission scenarios

**Figure 7:**
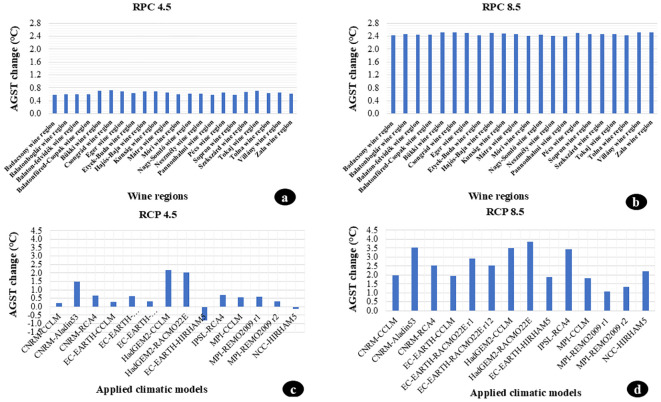
Projected changes in the Average Growing Season Temperature (AGST) between 2031 and 2100 based on different emission scenarios (RCP 4.5 and RCP 8.5) and the models used (14 models). **(A)** Projected changes in the Average Growing Season Temperature (AGST) for the 22 Hungarian wine regions between 2031 and 2100 based on the RCP 4.5 emission scenario. **(B)** Projected changes in the Average Growing Season Temperature (AGST) for the 22 Hungarian wine regions between 2031 and 2100 based on the RCP 8.5 emission scenario. **(C)** Projected changes in the Average Growing Season Temperature (AGST) based on 14 models between 2031 and 2100 under the RCP 4.5 emission scenario. **(D)** Projected changes in the Average Growing Season Temperature (AGST) based on 14 models between 2031 and 2100 under the RCP 8.5 emission scenario.

According to the more optimistic RCP 4.5 scenario, the average temperature during the vegetation period in Hungarian wine regions could rise by approximately 0.6-0.7°C over the next seven decades. The most significant increase in AGST (Average Growing Season Temperature) is expected in the Csongrád wine region. At the same time, the slightest change is anticipated in the Sopron wine region (**Figure 7A**). Under the more pessimistic RCP 8.5 scenario, the average temperature increase over the next seven decades is expected to exceed 2.0°C across Hungarian wine regions. The most significant growth, over 2.5°C, is expected in the Csongrád wine region. In contrast, the slightest change, 2.3°C, is expected in the Pannonhalma wine region (**Figure 7B**).

### 3.8 Temperature changes in Hungary according to different models based on various emission scenarios

The temperature differences due to the nature of specific climate models are significantly higher when considering regional differences. Under the RCP 4.5 scenario, the temperature differences between the models can reach up to 3°C. The highest average temperature during the vegetation period for the period 2031-2100 (2.2°C) is projected by the HadGEM2-CCLM model, while the EC-EARTH-HIRHAM5 model suggests a decrease in the average temperature during the vegetation period by 0.8°C over the next seven decades (**Figure 7C**). Under the RCP 8.5 scenario, the HadGEM2-RACMO22E model projects an average temperature increase of 3.8°C, whereas the MPI-REMO2009 r1 model projects that the average temperature during the vegetation period over the next seven decades will barely exceed 1.0°C (**Figure 7D**).

## 4 Discussion

Our results show that all of the bioclimatic indicators studied are clearly shifted towards higher temperature classes. We have plotted the spatial and temporal distribution of the change on a map, where the upward trend is observed throughout the country. However, significant regional differences emerge due to the effects of geographical and altitudinal zonality. For instance, wine regions in northern Hungary, such as Tokaj and Eger, exhibit higher rates of temperature increase compared to southern regions like Villány. These results will help develop different adaptation strategies for each wine-growing region for optimal varietal and technological change.

Every wine region has unique climatic conditions that determine which grape varieties are suitable for cultivation and what styles of wine can be produced in that region (Jones et al., 2010, 2012). If the climate changes, the style and character of wines produced in that region will also change (Santos et al., 2020). Therefore, it is essential to understand how climatic conditions change (Webb et al., 2008) and to what extent at a given location to take appropriate measures for quality grape cultivation (De Orduna, 2010; Neethling et al., 2012).

The temperature indices for Hungarian wine regions indicate a relatively narrow range, reflecting the climatic homogeneity of the region, in contrast to the broader range observed in New Zealand (Anderson, 2012). Moreover, these indices tend to be lower than those in Australian wine regions, highlighting Hungary’s relatively cooler climate (Hall and Jones, 2010). Jones et al. (2010) pointed out that these four examined temperature-based climate indicators and heat indexes provide numerical assistance for grape growers to determine which grape varieties have favourable climatic conditions at a given location. Our study also confirms the importance of these temperature indices in planning for future varietal changes, particularly in regions where AGST values are approaching or exceeding the upper thresholds for widely cultivated varieties like Merlot or Pinot Noir. These varieties are better adapted to warmer climates and can produce high-quality yields. However, it is unknown how these Mediterranean grape varieties, currently grown in Mediterranean regions, will develop in terms of aroma and flavour profiles under the unique microclimatic conditions of Hungarian growing sites.

There are differences in the climate data used in the climatic assessment of grape cultivation. Tonietto and Carbonneau (2004), in their study, alongside the Huglin Index (HI) (Huglin, 1978), also consider the Cool Night Index (Tonietto, 1999). This temperature-based index plays a crucial role in developing grape colour and aroma components (Kliewer and Torres, 1972; Kliewer, 1973; Tomana et al., 1979). Besides temperature indexes, examining water supply can also be a helpful indicator in suitability studies (Fraga et al., 2016). Drought poses a significant problem for European grape cultivation (Giorgi, 2006; Mariotti, 2010). Therefore, many vineyard suitability studies include water balance parameters, precipitation, and a Dryness Index (Riou et al., 1994). Incorporating these indices into future studies could enhance the understanding of how combined temperature and water stress factors impact Hungarian viticulture.

The results of the most pessimistic models, which show the most intense warming in wine regions, raise the question of how the yield of a given grape variety will be affected in years when the average temperature during the vegetation period exceeds 21°C. For example, in the case of Nebbiolo, which has a heat requirement of 18–21°C AGST, significant yield and quality losses could occur in Hungarian wine regions if AGST exceeds this range for extended periods. Numerous studies (Schultz, 2000; Jones et al., 2005, 2012; Duchêne and Schneider, 2005; Van Leeuwen and Darriet, 2016) confirm that the ripening processes of grapes accelerate in hot years. We do not have specific information on what exactly happens to the grapes if the average growing season temperatures exceed or fall short of the optimal intervals for the varieties. It can be assumed that metabolic disorders and heat or cold stress conditions are more likely to occur in such years. However, claiming that these grape varieties could not be cultivated in such years would be an exaggeration. High temperatures increase the photosynthesis and sugar formation in the grape berries (Greer and Weston, 2010; Palliotti et al., 2014), leading to a faster accumulation of sugars in the berries (Conde et al., 2007). However, high temperatures can negatively impact the biosynthesis of key secondary metabolites, such as anthocyanins and flavor compounds, which are essential for wine quality (Mori et al., 2007; Sadras and Moran, 2012). Simply advancing the harvest date may mitigate excessive sugar levels but fails to address the broader impacts of heat stress. To adapt to these challenges, a comprehensive approach combining vineyard management practices, enological strategies, and potentially breeding heat-tolerant grape varieties is required. This accelerated ripening poses a challenge for maintaining the balance of sugar, acidity, and phenolic compounds in grape berries, which is critical for wine quality.

Tóth and Végvári (2016) emphasise annual average temperature and temperature seasonality in their suitability study for grape cultivation in Europe, which aligns with our findings, showing that increasing temperatures will degrade the conditions for grape cultivation in the future. However, our results suggest that Hungarian wine regions may retain greater suitability for grapevine cultivation compared to Mediterranean regions, where severe droughts compound the effects of rising temperatures (Hannah et al., 2013). According to our suitability studies based on AGST, the expected changes in Hungarian vineyard sites by 2050 are only 2-17% according to the RCP 8.5 scenario. These results are significantly more favourable than the 25-73% reduction in suitability expected by 2050, according to Hannah et al. (2013), and much lower than the 2-48% decrease estimated by Tóth and Végvári (2016). Both studies cover the entire Mediterranean European region, where the average temperature during the vegetation period is higher than in Hungary. However, by the end of the century, the average temperature of Hungarian wine regions could exceed the current average temperatures of Southern European wine regions (Van Leeuwen et al., 2013). This underscores the need for long-term strategies that include introducing heat-tolerant grape varieties and adopting vineyard management practices to mitigate the effects of rising temperatures. The future increase in temperatures poses significant challenges for grape growers. On the one hand, they can cultivate new, higher heat-requiring varieties and clones. On the other hand, the performance and temperature response of previously cultivated lower heat-requiring varieties must be monitored and analysed with more outstanding care than before. Choosing the optimal harvest time will become increasingly important in future viticulture. Weather forecasts and their immediate incorporation into cultivation practices will become more crucial during ripening. Implementing measures to combat adverse weather effects will also become increasingly important. These measures may include frost protection, rain protection, or the use of regulated deficit irrigation (RDI) to mitigate drought effects.

## 5 Conclusions

### 5.1 Climate change and the shift in Hungarian viticulture

According to our study’s climate model results, certain Hungarian wine regions might experience vintages with average growing season temperatures exceeding 21°C in the future. This highlights the potential impacts of global warming on the suitability of winegrape varieties in these regions.The study confirmed that the temperature and heat indexes used to assess the suitability for grapevine cultivation show a definite increase in the near and distant future. The increase compared to historical values is expected to reach 25-50% in the distant future, indicating that ‘warm’ and ‘hot’ climatic conditions will become significantly more common in Hungarian wine regions.

### 5.2 Opportunities for new grape varieties

The biologically effective heat sum is expected to increase markedly, which indicates that it will be possible to cultivate grape varieties with higher heat requirements more safely in Hungarian wine regions. In the distant future, there will also be opportunities to cultivate grape varieties that are not currently grown and better tolerate high temperatures and stress, such as Grenache, Syrah, and Tempranillo.

### 5.3 Suitability of high heat-requirement varieties

The results show that grape varieties with higher heat requirements, such as Grenache, Carignane, Zinfandel, or Nebbiolo, will be safely cultivable in Hungarian wine regions even under the most optimistic scenarios. However, according to the most pessimistic scenario and models predicting the most significant temperature changes, even the higher heat-requiring grape varieties might experience a slight decrease in suitability by the end of the century in some Hungarian wine regions.

## Data Availability

The datasets used in this study are publicly available and can be accessed at the following website: https://nimbus.elte.hu/FORESEE/. This is an open-access database containing daily meteorological data with comprehensive descriptions.

## References

[B1] Anderson J. D. (2012). Analysis of viticulture region climate structure and suitability in New Zealand. OENO One 46, 149–165. doi: 10.20870/oeno-one.2012.46.3.1515

[B2] Bálo B. Katona Z. Olasz A. Tóth E. Deák T. Bodor P.. (2014). “Focus on terroir studies in the Eger wine region of Hungary,” in IVES Conference Series, Terroir. Available online at: https://ives-openscience.eu/wp-content/uploads/2020/07/B-lo-et-al_Focus-on-Terroir-Studies.pdf.

[B3] Conde C. Silva P. Fontes N. Dias A. C. P. Tavares R. M. Sousa M. J.. (2007). Biochemical changes throughout grape berry development and fruit and wine quality. Food Sci. Technol. Int. 13, 253–260.

[B4] Cook B. I. Wolkovich E. M. (2016). Climate change decouples drought from early wine grape harvests in France. Nat. Climate Change. 6 (7), 715–719. doi: 10.1038/nclimate2960

[B5] Czigány S. Novák T. J. Pirkhoffer E. Nagy G. Lóczy D. Dezső J.. (2020). Application of a topographic pedosequence in the Villány Hills for terroir characterization. Hungarian Geograph. Bull. 69, 1–17. doi: 10.15201/hungeobull.69.3.1

[B6] De Orduna R. M. (2010). Climate change associated effects on grape and wine quality and production. Food Res. Int. 43, 1844–1855. doi: 10.1016/j.foodres.2010.05.001

[B7] Dobor L. Barcza Z. Hlásny T. Havasi Á. Horváth F. Ittzés P.. (2014). Bridging the gap between climate models and impact studies: The FORESEE Database. Geosci. Data J. 2, 1–11. doi: 10.1002/gdj3.22 PMC5445562 28616227

[B8] Duchêne E. Huard F. Dumas V. Schneider C. Merdinoglu D. (2010). The challenge of adapting grapevine varieties to climate change. Climate Res. 41, 193–204. doi: 10.3354/cr00850

[B9] Duchêne E. Schneider C. (2005). Grapevine and climatic changes: a glance at the situation in Alsace. Agronomy for Sustainable Development 25 (1), 93–99. doi: 10.1051/agro:2004057

[B10] Dunn M. Rounsevell M. D. Boberg F. Clarke E. Christensen J. Madsen M. S. (2019). The future potential for wine production in Scotland under high-end climate change. Region. Environ. Change 19, 723–732. doi: 10.1007/s10113-017-1240-3

[B11] Fazekas I. Nyitrainé-Sárdy Á.,D. Taranyi D. Varga Zs. (2022). A hagyományos szőlőműveléshez köthető borszőlő-fajták területi aránya Magyarországon 2015-ben és 2020-ban. Horticulture/Kertgazdaság 54, 29. doi: 10.5772/64976

[B12] Fraga H. Malheiro A. C. Moutinho-Pereira J. Santos J. A. (2012). An overview of climate change impacts on European viticulture. Food Energy Secur. 1, 94–110. doi: 10.1002/fes3.2013.1.issue-2

[B13] Fraga H. Santos J. A. Malheiro A. C. Oliveira A. A. Moutinho-Pereira J. Jones G. V. (2016). Climatic suitability of Portuguese grapevine varieties and climate change adaptation. Int. J. Climatol. 36, 1–12. doi: 10.1002/joc.4325

[B14] Gannon K. E. Conway D. Hardman M. Nesbitt A. Dorling S. Borchert J. (2023). Adaptation to climate change in the UK wine sector. Climate Risk Manage. 42, 100572. doi: 10.60692/aas3e-3tc85

[B15] García de Cortázar-Atauri I. Duchêne E. Destrac-Irvine A. Barbeau G. de Rességuier L. Lacombe T.. (2017). Grapevine phenology in France: from past observations to future evolutions in the context of climate change. OENO One 51, 115–126. doi: 10.20870/oeno-one.2017.51.2.1622

[B16] Giorgi F. (2006). Climate change hot spots. Geophys. Res. Lett. 33, L08707. doi: 10.1029/2006GL025734

[B17] Gladstones J. (1992). Viticulture and Environment. Winetitles, Adelaide, 1–310.

[B18] Greer D. H. Weston C. (2010). Effects of fruiting on vegetative growth and development dynamics of grapevines (Vitis vinifera cv. Semillon) can be traced back to events at or before budbreak. Funct. Plant Biol. 37, 756–766. doi: 10.1071/FP09297

[B19] Gustafsson J. G. Mårtensson A. (2005). Potential for extending Scandinavian wine cultivation. Acta Agricult. Scandinavica Section B-Soil Plant Sci. 55, 82–97. doi: 10.1080/09064710510029097

[B20] Hajdu E. (2018). Viticulture of Hungary. Acta Agraria Debreceniensis 150, 175–182. doi: 10.34101/actaagrar/150/1713

[B21] Hall A. Jones G. V. (2010). Spatial analysis of climate in winegrape-growing regions in Australia. Aust. J. Grape Wine Res. 16, 389–404. doi: 10.1111/j.1755-0238.2010.00100.x

[B22] Hannah L. Roehrdanz P. R. Ikegami M. Shepard A. V. Shaw M. R. Tabor G.. (2013). Climate change, wine, and conservation. Proc. Natl. Acad. Sci. 110, 6907–6912. doi: 10.1073/pnas.1210127110 23569231 PMC3637704

[B23] Hlédik E. Harsányi D. (2019). Wine tourism in Hungary–wine and destination preferences of wine tour participants. In 18th International Congress of the International Association on Public and Nonprofit Marketing, Conference Proceeding, “Challenges in Public, Non-Profit and Social Marketing”. Ercsey I. Réka K. Eds. Department of Marketing and Management, University of Győr, H-9026 Győr, Egyetem tér 1., 45–57. Available online at: https://uni.sze.hu.

[B24] Huglin P. (1978). Nouveau mode d’évaluation des possibilités héliothermiques de la vigne. C.R. Acad. Agric. Fr 64 (13), 1117–1126.

[B25] IPCC CoreWriting Team (2014). “Climate Change 2014: Synthesis Report,” in Contribution of Working Groups I, II and III to the Fifth Assessment Report of the Intergovernmental Panel on Climate Change. Eds. Pachauri R. K. Meyer L. A. (IPCC, Geneva, Switzerland), 151.

[B26] Jackson R. S. (2008). Wine Science: Principles and Applications (Burlington, MA, USA: Academic Press)., 377.

[B27] Jobin Poirier E. Plummer R. Pickering G. (2021). Climate change adaptation in the Canadian wine industry: Strategies and drivers. Can. Geographer/Le Géographe canadien 65, 368–381. doi: 10.1111/cag.12665

[B28] Jones G. V. (2006a). “Climate and Terroir: Impacts of Climate Variability and Change on Wine,” in Fine Wine and Terroir - The Geoscience Perspective, vol. 247 . Eds. Macqueen R. W. Meinert L. D. (Geological Association of Canada, St. John’s, Newfoundland).

[B29] Jones G. V. (2006b). Climate and terroir: impacts of climate variability and change on wine. Geosci. Canada Reprint Ser. 9, 203–217.

[B30] Jones G. V. (2006c). Climate change and wine: Observations, impacts and future implications. Wine Industry J. 21, 21–26.

[B31] Jones G. (2015). Climate, Grapes, and Wine. Terroir and the importance of climate on grapevine production. GuildSomm. Available at: https://www.guildsomm.com/public_content/features/articles/b/gregory_jones/posts/climategrapes-and-wine (Accessed July 17, 2024).

[B32] Jones G. V. Davis R. E. (2000). Climate influences on grapevine phenology, grape composition, and wine production and quality for Bordeaux, France. Am. J. Enol Vitic 51, 249–261. doi: 10.5344/ajev.2000.51.3.249

[B33] Jones G. V. Duff A. A. Hall A. Myers J. W. (2010). Spatial analysis of climate in winegrape growing regions in the western United States. Am. J. Enol. Viticult. 61, 313–326. doi: 10.5344/ajev.2010.61.3.313

[B34] Jones G. V. Reid R. Vilks A. (2012). “Climate, Grapes, and Wine: Structure and Suitability in a Variable and Changing Climate,” in The Geography of Wine: Regions, Terroir, and Techniques. Ed. Dougherty P. (Dordrecht: Springer Press), 255. doi: 10.1007/978-94-007-0464-0_7

[B35] Jones G. V. White M. A. Cooper O. R. Storchmann K. (2005). Climate change and global wine quality. Climatic Change 73, 319–343. doi: 10.1007/s10584-005-4704-2

[B36] Keller M. (2010). The Science of Grapevines: Anatomy and Physiology (Burlington, MA, USA: Academic Press).

[B37] Kern A. Dobor L. Hollós R. Marjanovic H. Torma C. Kis A.. (2024). Seamlessly combined historical and projected daily meteorological datasets for impact studies in Central Europe: the FORESEE v4.0 and the FORESEE-HUN v1.0. Climate Serv. 33, 100443. doi: 10.1016/j.cliser.2023.100443

[B38] Kliewer W. M. (1973). Berry composition of *Vitis vinifera* cultivars as influenced by photo and nycto-temperatures during maturation. J. Am. Soc Hortic. Sci. 2, 153–159. doi: 10.21273/JASHS.98.2.153

[B39] Kliewer W. M. Torres R. E. (1972). Effect of controlled day and night temperatures on grape coloration. Am. J. Enol.Vitic. 2, 71–77. doi: 10.5344/ajev.1972.23.2.71

[B40] Kunicka-Styczyńska A. Czyżowska A. Ratajkowska K. Witkowska A. Dziugan P. (2016). The trends and prospects of winemaking in Poland. Grape Wine Biotechnol., 401–413. doi: 10.5772/64976

[B41] Lakatos L. Mika J. (2022). Analysis of quadratic correlation between dryness indices and wine grape yield to estimate future climate impacts in hungary. Climate 10 (11), 165. doi: 10.3390/cli10110165

[B42] Malheiro A. C. Santos J. A. Fraga H. Pinto J. G. (2010). Climate change scenarios applied to viticultural zoning in Europe. Climate Res. 43, 163–177. doi: 10.3354/cr00918

[B43] Mariotti A. (2010). Recent changes in the Mediterranean water cycle: A pathway toward long-term regional hydroclimatic change? J. Climate 23, 1513–1525. doi: 10.1175/2009JCLI3251.1

[B44] Masson-Delmotte V. Zhai P. Pirani A. Connors S. L. Péan C. Berger S.. (2021). Climate Change 2021: The Physical Science Basis. Contribution of Working Group I to the Sixth Assessment Report of the Intergovernmental Panel on Climate Change. Cambridge University Press, Cambridge, UK, and New York, NY, USA. 2(1), 2391.

[B45] Mód L. Simon A. (2012). Wine districts, wine regions, vineyards—the construction and representation of borders in the Hungarian wine culture. Acta Ethnogr. Hungarica 57, 75–90. doi: 10.1556/AEthn.57.2012.1.7

[B46] Mori K. Goto-Yamamoto N. Kitayama M. Hashizume K. (2007). Loss of anthocyanins in red-wine grape under high temperature. J. Exp. Bot. 58, 1935–1945. doi: 10.1093/jxb/erm055 17452755

[B47] Mullins M. G. Bouquet A. Williams L. E. (1992). Biology of the Grapevine (Cambridge, UK: Cambridge University Press).

[B48] Nakicenovic N. Alcamo J. Davis G. de Vries B. Fenhann J. Gaffin S.. (2000). IPCC special report on emissions scenarios (Cambridge: Cambridge University Press).

[B49] Neethling E. Barbeau G. Bonnefoy C. Quenol H. (2012). Change in climate and berry composition for grapevine varieties cultivated in the Loire Valley. Clim. Res. 53, 89–101. doi: 10.3354/cr01094

[B50] Németh E. Horváth I. Bidló A. Hofmann T. (2014). Evaluating soil, grape and wine composition in the Sopron Wine Region, Hungary. Agrokémia és Talajtan 63, 165–174. doi: 10.1556/agrokem.63.2014.1.18

[B51] Novák T. J. Hegyi B. Balogh S. Czímer B. Rózsa P. (2023). How geoecological components of a terroir can be altered by spatial changes of vineyards–A case study from Eger Wine District (Hungary). Erdkunde 77 (3), 213–232.

[B52] Palliotti A. Tombesi S. Silvestroni O. Lanari V. Gatti M. Poni S. (2014). Changes in vineyard establishment and canopy management urged by earlier climate-related grape ripening: a review. Scientia Hortic. 178, 43–54. doi: 10.1016/j.scienta.2014.07.039

[B53] Rauhut Kompaniets O. (2022). Sustainable competitive advantages for a nascent wine country: an example from southern Sweden. Competitive. Review: Int. Business J. 32, 376–390. doi: 10.1108/CR-04-2021-0063

[B54] Riou Ch. Becker N. Sotes Ruiz V. Gomez-Miguel V. Carbonneau A. Panagiotou M.. (1994). Le déterminisme climatique de la maturation du raisin: application au zonage de la teneur em sucre dans la communauté européenne (Luxembourg, 322: Office des Publications Officielles des Communautés Européennes).

[B55] Sadras V. O. Moran M. A. (2012). Elevated temperature decouples anthocyanins and sugars in berries of Shiraz and Cabernet Franc. Aust. J. Grape Wine Res. 18, 115–122. doi: 10.1111/j.1755-0238.2012.00180.x

[B56] Santos J. A. Fraga H. Malheiro A. C. Moutinho-Pereira J. Dinis L. T. Correia C.. (2020). A review of the potential climate change impacts and adaptation options for European viticulture. Appl. Sci. 10, 3092. doi: 10.3390/app10093092

[B57] Santos J. A. Malheiro A. C. Karremann M. K. Pinto J. G. (2010). Climate change scenarios for viticultural zoning in Europe. Climatic Change 98, 31–42. doi: 10.3354/cr00918

[B58] Schernewski G. (2011). “Adaptation to climate change: viniculture and tourism at the Baltic Coast,” in Global change and Baltic coastal zones (Springer Netherlands, Dordrecht), 233–247.

[B59] Schultz H. R. (2000). Climate change and viticulture: a European perspective on climatology, carbon dioxide, and UV-B effects. Aust. J. Grape Wine Res. 6, 2–12. doi: 10.1111/j.1755-0238.2000.tb00156.x

[B60] Schultz H. R. Jones G. V. (2010). : climate induced historic and future changes in viticulture. J. Wine Res. 21, 137–145. doi: 10.1080/09571264.2010.530098

[B61] Szabó G. Závodi B. (2018). The tourism geographical characteristics of wine gastronomy festivals in the Balaton Wine Region. Pannon Manage. Rev. 7, 27–43.

[B62] Szenteleki K. Ladányi M. Gaál M. Zanathy G. Bisztray G. Y. (2012). Climatic risk factors of Central Hungarian grape growing regions. Appl. Ecol. Environ. Res. 10, 87–105. doi: 10.15666/aeer/1001_087105

[B63] Szepesi J. Harangi S. Ésik Z. Novák T. J. Lukács R. Soós I. (2017). Volcanic geoheritage and geotourism perspectives in Hungary: A case of an UNESCO world heritage site, Tokaj wine region historic cultural landscape, Hungary. Geoheritage 9, 329–349. doi: 10.1007/s12371-016-0205-0

[B64] Szivas E. (1999). The development of wine tourism in Hungary. Int. J. Wine Market. 11, 7–17. doi: 10.1108/eb008692

[B65] Tomana T. Utsunomiya N. Kataoka I. (1979). The effect of environmental temperatures on fruit on ripening on the tree. II. The effect of temperatures around whole vines and clusters on the coloration of ‘Kyoho’grapes. J. Jap. Soc Hortic. Sci. 48, 261–266. doi: 10.2503/jjshs.48.261

[B66] Tonietto J. Carbonneau A. (2004). A multicriteria climatic classification system for grape-growing regions worldwide. Agric. For. Meteorol. 124, 81–97. doi: 10.1016/j.agrformet.2003.06.001

[B67] Tonietto J. (1999). Les macroclimats viticoles mondiaux et l’influence du mesoclimat sur la typicité de la Syrah et du Muscat de Hambourg dans le sud de la France: méthodologie de caractérisation. Doctoral dissertation. École Nationale Supérieure Agronomique de Montpellier, Formation Doctorale - Biologie de l’Évolution et Écologie, Montpellier, France.

[B68] Tóth J. P. Végvári Z. (2016). Future of winegrape growing regions in Europe. Aust. J. Grape Wine Res. 22 (1), 64–72. doi: 10.1111/ajgw.12168

[B69] Van Leeuwen C. Darriet P. (2016). The impact of climate change on viticulture and wine quality. J. Wine Econ. 11, 150–167. doi: 10.1017/jwe.2015.21

[B70] Van Leeuwen C. Friant P. Chone X. Tregoat O. Koundouras S. Dubourdieu D. (2004). Influence of climate, soil, and cultivar on terroir. Am. J. Enol. Viticult. 55, 207–217. doi: 10.5344/ajev.2004.55.3.207

[B71] Van Leeuwen C. Schultz H. R. Garcia de Cortazar-Atauri I. Duchêne E. Ollat N. Pieri P.. (2013). Why climate change will not dramatically decrease viticultural suitability in main wine-producing areas by 2050. Proc. Natl. Acad. Sci. 110, E3051–E3052. doi: 10.1073/pnas.1307927110 23792579 PMC3746856

[B72] Van Leeuwen C. Seguin G. (2006). The concept of terroir in viticulture. J. Wine Res. 17, 1–10. doi: 10.1080/09571260600633135

[B73] Webb L. B. Whetton P. H. Barlow E. W. R. (2008). Modelling the relationship between climate, winegrape price and winegrape quality in Australia. Clim. Res. 36, 89–98. doi: 10.3354/Cr00739

[B74] Winkler A. J. Cook J. A. Kliewer W. M. Lider L. A. (1974). General Viticulture. 4th Edition (Berkeley: University of California Press), 740.

[B75] Ziernicka-Wojtaszek A. Zawora T. (2007). Global warming and grapevine cultivation opportunities in Poland. In Réchauffement climatique, quels impacts probables sur les vignobles? / Global Warming: Which Potential Impacts on the Vineyards?, March 28–30, 2007. Department of Meteorology and Climatology, University of Agriculture, Cracow, al. Mickiewicza 24/28, Poland, 28–30.

